# Ultrasound-Assisted Extraction of Antioxidant Polyphenols from Zheng’an White Tea: Process Optimization and Bioactivity

**DOI:** 10.3390/foods15183281

**Published:** 2026-09-17

**Authors:** Yucheng He, Jun Liu, Ran Ye, Xiang Zheng, Yuchun Rao, Fang Wang

**Affiliations:** 1 Xingzhi College, Zhejiang Normal University, Jinhua 321004, China; hyc585058@163.com; 2 College of Life Sciences, Zhejiang Normal University, Jinhua 321004, China; 18267889248@163.com (J.L.); 18368752810@163.com (R.Y.); 1213372503@zjnu.edu.cn (X.Z.)

**Keywords:** Zheng’an white tea, tea polyphenols, ultrasound-assisted extraction, response surface methodology, antioxidant activity

## Abstract

White tea is rich in polyphenols with antioxidant potential, but information on Zheng’an white tea remains limited. This study optimized water-based ultrasound-assisted extraction (UAE) using single-factor experiments and a Box–Behnken design and evaluated the antioxidant activity of the resulting extract. Tea-leaf microstructure, polyphenolic composition, and antioxidant capacity were assessed using scanning electron microscopy (SEM), liquid chromatography–mass spectrometry (LC–MS), in vitro radical-scavenging assays, and a *Caenorhabditis elegans* model, respectively. The optimized conditions within the investigated experimental domain were a solid-to-liquid ratio of 1:50 (g/mL), an ultrasonic time of 50 min, and an ultrasonic power of 118 W, yielding a total polyphenol content of 37.93 ± 0.17 mg GAE/g DW. SEM revealed marked disruption of tea-leaf cellular structures after UAE. LC–MS enabled the putative annotation of 25 representative polyphenols spanning phenolic acids, flavan-3-ols, procyanidins, flavonols and their glycosides, and related derivatives. At 1.5 mg/mL, the extract exhibited ABTS and DPPH radical-scavenging rates of 81.68 ± 3.23% and 85.43 ± 2.31%, respectively. In *C. elegans*, 100 μg/mL extract increased catalase activity by 86.03% and decreased malondialdehyde content by 46.40% (*p* < 0.01). These findings show that the optimized aqueous UAE procedure recovered a polyphenol-rich extract with antioxidant activity in the tested chemical assays and *C. elegans* model.

## 1. Introduction

Tea (*Camellia sinensis* (L.) O. Kuntze) is one of the most widely consumed beverages worldwide and has attracted considerable attention because of its nutritional value and health-promoting properties. Among the diverse bioactive constituents of tea, tea polyphenols represent the major functional components responsible for many of its biological activities, including antioxidant [1], anti-inflammatory [2], antimicrobial [3], and cardiovascular protective effects [4]. Owing to these beneficial properties, tea polyphenols have been extensively investigated for applications in functional foods, nutraceuticals, and natural antioxidant products. Recent advances in extraction, purification, and analytical technologies have further promoted the efficient utilization of tea polyphenols in food and pharmaceutical industries.

Zheng’an white tea, produced from the temperature-sensitive albino tea cultivar “Baiye No. 1”, develops pale tender leaves under low-temperature conditions and is valued for the distinctive chemical and sensory characteristics associated with albino tea shoots [5]. Although green tea [6], black tea [7], and white tea [8] have been studied extensively, information specifically concerning the extraction and antioxidant properties of Zheng’an white tea remains limited. Therefore, developing efficient strategies for recovering its bioactive compounds is of considerable importance for improving its value-added utilization and promoting the development of functional tea products.

Conventional extraction methods for tea polyphenols, such as hot-water extraction, solvent reflux extraction, and Soxhlet extraction, often suffer from long extraction times, high solvent consumption, and the risk of thermal degradation of thermolabile compounds [9,10]. UAE can enhance solvent penetration, disrupt plant tissues, and accelerate mass transfer through acoustic cavitation, thereby improving extraction efficiency [11,12]. Its performance depends on operating variables including ultrasonic power, ultrasonic time, solvent-to-solid ratio, and temperature. Excessive ultrasonic power or prolonged treatment may expose released phenolics to localized thermal and sonochemical effects; consequently, optimization is required to balance mass transfer against possible degradation [13,14,15].

Response surface methodology (RSM) has become one of the most widely used statistical tools for optimizing extraction processes because it enables simultaneous evaluation of multiple experimental variables and their interactions while minimizing the number of experimental runs. Numerous studies have successfully applied RSM to optimize UAE conditions for various tea products and medicinal plants, leading to significant improvements in total phenolic recovery and antioxidant capacity [16,17]. Meanwhile, the rapid development of liquid chromatography-mass spectrometry (LC-MS) has greatly facilitated the comprehensive characterization of plant polyphenols [18], providing valuable information for elucidating the relationship between phytochemical composition and biological activity.

Although UAE optimization and phytochemical profiling have been reported separately for several tea materials [19,20], an integrated evaluation combining aqueous UAE optimization, LC–MS annotation, chemical antioxidant assays, and a *C. elegans* model has not been reported for Zheng’an white tea. The present study addresses this cultivar-specific knowledge gap while recognizing that comparisons with conventional extraction, alternative solvent systems, and lower-value tea materials require separate matched experimental designs.

Therefore, this study aimed to optimize three aqueous UAE variables—solid-to-liquid ratio, ultrasonic time, and ultrasonic power—using a Box–Behnken response surface design; examine ultrasound-associated microstructural changes by SEM; putatively annotate representative polyphenols by LC–MS; and evaluate antioxidant responses using ABTS/DPPH assays and a *C. elegans* model. The resulting evidence is intended to provide a laboratory-scale basis for future evaluation of Zheng’an white tea extracts in food-oriented applications.

## 2. Materials and Methods

### 2.1. Plant Material, Nematodes, and Reagents

Zheng’an white tea (*Camellia sinensis* (L.) O. Kuntze, cultivar “Baiye No. 1”) was obtained from Zheng’an, Guizhou Province, China. The N2 wild-type strain of *Caenorhabditis elegans* and *Escherichia coli* OP50 were obtained from Zhejiang Normal University.

Gallic acid, Folin–Ciocalteu reagent, 2,2-diphenyl-1-picrylhydrazyl (DPPH), and 2,2′-azino-bis(3-ethylbenzothiazoline-6-sulfonic acid) (ABTS) were purchased from Beijing Solarbio Science & Technology Co., Ltd. (Beijing, China). Sodium carbonate, methanol, LC-MS-grade methanol, formic acid, and other analytical-grade reagents were obtained from Sinopharm Chemical Reagent Co., Ltd. (Shanghai, China). Catalase (CAT) and malondialdehyde (MDA) assay kits were purchased from Nanjing Jiancheng Bioengineering Institute (Nanjing, China).

### 2.2. Apparatus

The apparatus included an analytical balance (Practum224-1CN, Sartorius Scientific Instruments (Beijing) Co., Ltd., Beijing, China), a UV–visible spectrophotometer (T6 New Century, Beijing Purkinje General Instrument Co., Ltd., Beijing, China), a constant-temperature ultrasonic cleaner (LC-TUC-150, Lichen Scientific Instruments (Zhejiang) Co., Ltd., Shaoxing, Zhejiang, China), a grinder (AF-10SA, Aoli Traditional Chinese Medicine Machinery Co., Ltd., Wenling, Zhejiang, China), a rotary evaporator (RE-52AA, Shanghai Yarong Instrument Co., Ltd., Shanghai, China), a freeze dryer (YTLC-10A, Shanghai Yetuo Technology Co., Ltd., Shanghai, China), a constant-temperature incubator (LRH-70, Shanghai Yiheng Scientific Instruments Co., Ltd., Shanghai, China), an ion sputter coater (E-1030, Hitachi High-Technologies Corporation, Tokyo, Japan), a scanning electron microscope (GeminiSEM 300, Carl Zeiss Microscopy GmbH, Jena, Germany), a high-speed refrigerated centrifuge (Centrifuge 5810 R, Eppendorf AG, Hamburg, Germany), and an UltiMate 3000 UHPLC coupled to a Q Exactive hybrid quadrupole-Orbitrap mass spectrometer (Thermo Fisher Scientific, Waltham, MA, USA).

### 2.3. Preparation of Tea Powder

Fresh Zheng’an white tea leaves were washed with distilled water, air-dried, coarsely cut with scissors, and then pre-frozen in the cold trap of a vacuum freeze dryer at −60 °C for 4 h. The prefrozen leaves were spread evenly on drying shelves. When the cold trap temperature reached −40 °C, the vacuum was applied. After the chamber pressure dropped below 10 Pa, the samples were freeze-dried for 12 h. The lyophilized leaves were then ground in a mill for 10 s and passed through an 80-mesh sieve (aperture ≤ 180 μm). The resulting powder was sealed and stored at 4 °C until use.

### 2.4. Single-Factor Experiments

Ultrasound-assisted extraction was performed in a constant-temperature ultrasonic bath (fixed frequency, 40 kHz; tank volume, 15 L). For each extraction, 0.500 g of tea powder was mixed with ultrapure water at the desired solid-to-liquid ratio in a beaker. The beaker was placed in the bath without contacting the tank walls, and the bath-water level was maintained above the sample-liquid level. The temperature was set to 40 °C based on a previous UAE study of tea showing favorable polyphenol recovery at this temperature [21] and controlled by the built-in probe, with a calibrated thermometer immersed below the bath-water surface for auxiliary monitoring. Ultrasonic power was set using the instrument controls. The effects of solid-to-liquid ratio (1:20, 1:40, 1:60, 1:80, and 1:100 g/mL), ultrasonic irradiation time (30, 45, 60, 75, and 90 min), and ultrasonic power (80, 110, 140, 170, and 200 W) on the extraction yield were investigated. Unless otherwise specified, the baseline conditions were as follows: solid-to-liquid ratio, 1:60 g/mL; ultrasonic irradiation time, 60 min; ultrasonic power, 140 W; and bath temperature, 40 °C. The delivered acoustic power was not independently measured; according to the manufacturer, the instrument output had been factory calibrated using an ultrasonic power meter and voltage/current testing.

### 2.5. Box–Behnken Experimental Design

Based on the single-factor results, a three-factor, three-level Box–Behnken design (BBD) was used [22] to evaluate the effects of solid-to-liquid ratio (A), ultrasonic time (B), and ultrasonic power (C) on total polyphenol content (TPC). The factor levels are presented in Table 1.

### 2.6. Determination of Total Polyphenol Content (TPC)

TPC was determined using the Folin–Ciocalteu assay with gallic acid as the calibration standard [23]. The ultrasonic-treated extract was centrifuged at 8000 rpm for 10 min, and the supernatant was collected and diluted. A 2 mL aliquot of the diluted supernatant was mixed with 0.5 mL of 10% (*v*/*v*) Folin–Ciocalteu reagent and vortexed for 30 s. Then, 2 mL of 12% (*w*/*v*) sodium carbonate solution was added, and the mixture was made up to 25 mL with distilled water. The solution was incubated in the dark at room temperature for 2 h. The absorbance was measured at 760 nm. The dilution factor was adjusted so that the measured absorbance values fell within the range of 0.1–1.0, ensuring that the measurements were within the linear range.

TPC = C × V × DF/m

where C is the gallic-acid-equivalent concentration obtained from the calibration curve (mg/mL), V is the total extraction volume (mL), DF is the dilution factor, and m is the dry mass of tea powder (g). Results are expressed as mg gallic acid equivalents per g dry weight (mg GAE/g DW).

### 2.7. Scanning Electron Microscopy Analysis

Tea powder samples were mounted on conductive carbon tape and sputter-coated with gold at 30 mA for 30 s. The samples were observed using a SEM equipped with a secondary electron detector at an accelerating voltage of 5 kV and a working distance of 5 mm. The surface morphology of untreated, water-immersed, and ultrasonically treated samples was compared.

### 2.8. Polyphenolic Composition Analysis

The LC–MS workflow followed Hu et al. [24]. The extract obtained under the optimized conditions was centrifuged at 8000 rpm for 10 min, and the resulting supernatant was collected. A 10 mL aliquot of the supernatant was concentrated to near dryness by rotary evaporation at 45 °C under vacuum (−0.1 MPa) and subsequently reconstituted in 300 μL of methanol. The reconstituted solution was filtered through a 0.22 μm hydrophilic polyvinylidene fluoride membrane filter before LC–MS analysis. Chromatographic separation was performed on an ACQUITY Premier HSS T3 column (1.8 μm, 2.1 × 100 mm) maintained at 45 °C. The mobile phases consisted of water containing 0.1% formic acid (A) and methanol (B), and the gradient elution program is presented in Table 2. Mass spectrometric data were acquired separately in positive- and negative-ion modes using Xcalibur software (version 4.3) under data-dependent acquisition (DDA). Full-scan MS spectra were acquired over an *m*/*z* range of 150–1500 at a resolution of 70,000, with an automatic gain control (AGC) target of 1 × 10^6^ and a maximum injection time of 50 ms. The ten most intense precursor ions in each full scan were selected for data-dependent MS/MS analysis. MS/MS spectra were acquired at a resolution of 17,500, with an AGC target of 1 × 10^5^ and a maximum injection time of 50 ms, using stepped normalized collision energies of 10, 30, and 55. Raw MS files were converted to mzXML format using MSConvert in ProteoWizard (version 3.0.8789). Raw data were preprocessed using the XCMS package in R, including peak detection, peak filtering, and peak alignment, with the following parameters: bw = 2, ppm = 15, peakwidth = c(5, 30), mzwid = 0.015, mzdiff = 0.01, and method = “centWave”. Metabolite annotation was subsequently performed using the metID package by matching the aligned features against public databases, including HMDB, MassBank, LIPID MAPS, mzCloud, and KEGG. MS1 mass accuracy, retention time, and MS/MS spectral similarity were integrated into a total matching score. The precursor-ion forms considered during annotation included [M+H]^+^, [M−H_2_O+H]^+^, and [2M+H]^+^ in positive-ion mode, and [M−H]^−^, [2M−H]^−^, and [M+HCOO]^−^ in negative-ion mode. The precursor-ion mass tolerance was set to <15 ppm. Retention times (RTs) were recorded for all detected features and are provided in the annotation table; RT was used as supporting evidence within the total-score calculation rather than as an independent criterion for metabolite identification. Because no authentic standards were used for confirmation, all identified metabolites were reported as putatively annotated compounds.

### 2.9. In Vitro Free Radical Scavenging Assays

The extract obtained under the optimal conditions was centrifuged at 8000 rpm for 10 min, and the supernatant was collected. The supernatant was concentrated to one-third of its original volume using a rotary evaporator under reduced pressure at a water bath temperature of 45 °C and a vacuum degree of −0.1 MPa. The concentrated solution was then pre-frozen in the cold trap of the freeze dryer at −60 °C for 4 h. After pre-freezing, the samples were transferred to the drying shelves of the freeze dryer. The shelf temperature was set at −20 °C, and the vacuum was applied until the pressure fell below 10 Pa. The samples were lyophilized for 10 h to obtain the tea extract powder. The powder was then reconstituted at the required concentrations to prepare the sample solutions. The DPPH and ABTS radical-scavenging activities of the sample solutions were determined using spectrophotometric methods according to Sarker et al. [25], with minor modifications.

For the DPPH assay, 10 μL of the sample solution was mixed with 1.0 mL of freshly prepared DPPH solution (250 μM), followed by the addition of 4.0 mL of ethanol. The reaction mixture was incubated in the dark at room temperature for 30 min, after which its absorbance was measured at 517 nm. Each measurement was performed in triplicate.

For the ABTS assay, ABTS (7.4 mM) and potassium persulfate (2.6 mM) stock solutions were prepared separately and mixed in equal volumes. The mixture was allowed to react in the dark at room temperature for 12 h to generate the ABTS radical cation (ABTS•+). Before use, the resulting ABTS•+ solution was diluted with methanol at a ratio of 1:60 (*v*/*v*) to prepare the working solution. Subsequently, 150 μL of the sample solution was mixed with 2.85 mL of the ABTS•+ working solution and incubated in the dark at room temperature for 2 h. The absorbance was then measured at 734 nm against methanol. Each measurement was performed in triplicate. Vitamin C (VC) was used as the reference antioxidant where indicated. Radical-scavenging activity was calculated using the following equation:

Radical scavenging activity (%) = [1 − (Ai − Aj)/A0] × 100

Here, A0 is the absorbance of the blank control, Ai is the absorbance of the sample group, and Aj is the absorbance of the sample blank.

### 2.10. In Vivo Antioxidant Assay in Caenorhabditis elegans

The in vivo antioxidant activity of the tea extract was evaluated using wild-type N2 *Caenorhabditis elegans* according to Xu et al. [26], with modifications. The nematodes were maintained at 20 °C on nematode growth medium (NGM) plates seeded with *Escherichia coli* OP50 as the food source. Age-synchronized populations were obtained using the sodium hypochlorite synchronization method.

Synchronized L4-stage nematodes were transferred to NGM plates containing tea extract at final concentrations of 10, 50, or 100 μg/mL and incubated at 20 °C for 48 h. NGM plates without tea extract served as the untreated control. Approximately 500 nematodes from each treatment group were collected and washed three times with M9 buffer to remove residual OP50 and tea extract. The nematodes were then resuspended in an appropriate volume of M9 buffer and disrupted in an ice bath. The resulting homogenates were centrifuged at 3000 rpm for 10 min, and the supernatants were collected for biochemical analysis.

Catalase (CAT) activity and malondialdehyde (MDA) content were determined using the corresponding commercial assay kits according to the manufacturer’s instructions. The protein concentration of each supernatant was determined using a bicinchoninic acid assay, and CAT activity and MDA content were normalized to the protein concentration and expressed as U/mg protein and nmol/mg protein, respectively. Three independent biological replicates were performed for each treatment.

### 2.11. Statistical Analysis

Statistical analyses were performed using SPSS Statistics 26.0 (IBM Corp., Armonk, NY, USA). The single-factor optimization experiments, validation of the optimal response surface conditions, and antioxidant activity assays were all performed with three independent replicates. Data are presented as the mean ± standard deviation (SD). Differences among groups were evaluated using one-way analysis of variance (ANOVA), followed by Tukey’s honestly significant difference (HSD) post hoc test for multiple comparisons. Differences were considered statistically significant at (*p* < 0.05) and highly significant at (*p* < 0.01). Figures were generated using Origin 2023 (OriginLab Corporation, Northampton, MA, USA). The Box–Behnken experimental design, regression-model fitting, analysis of variance, and optimization of the response-surface model were performed using Design-Expert 13 (Stat-Ease Inc., Minneapolis, MN, USA).

## 3. Results

### 3.1. Single-Factor Experimental Results

**Figure 1:**
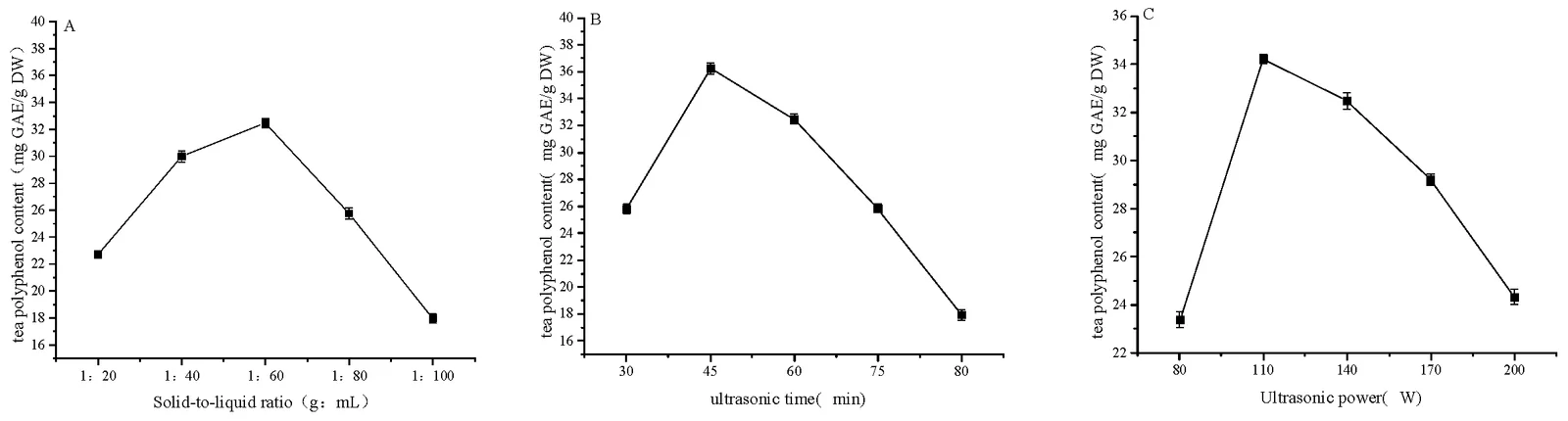
Effects of solid-to-liquid ratio (**A**), ultrasonic time (**B**), and ultrasonic power (**C**) on total polyphenol content.

The solid-to-liquid ratio, ultrasonic time, and ultrasonic power influenced TPC under the tested conditions. As shown in Figure 1A, TPC increased to 32.47 ± 0.25 mg GAE/g DW at 1:60 g/mL and then decreased slightly at higher solvent volumes. The initial increase is consistent with improved concentration-gradient-driven mass transfer; however, the cause of the subsequent decline was not measured and should not be attributed to co-extracted impurities without direct evidence [13].

TPC reached 36.25 ± 0.42 mg GAE/g DW at 45 min (Figure 1B) and decreased at longer treatment times. Prolonged sonication may expose released phenolics to thermal or sonochemical changes [14], but degradation products were not measured in this study; this mechanism therefore remains inferential.

At an ultrasonic power of 110 W, TPC reached 34.23 ± 0.19 mg GAE/g DW (Figure 1C). Higher settings produced lower TPC values. Although stronger cavitation can alter plant tissues and mass transfer, the present data do not distinguish thermal degradation from other possible causes [15].

### 3.2. Response Surface Optimization

To evaluate the interaction effects of these variables and pinpoint the optimal extraction conditions, a three-factor, three-level BBD was executed. The experimental design and corresponding results are detailed in Table 3. The relationship between the TPC (Y) and the coded independent variables (solid-to-liquid ratio, A; time, B; power, C) was established via the following second-order polynomial equation:

Y = 37.09 − 1.56A + 2.07B + 2.02C − 1.04AB − 1.38AC + 1.14BC − 2.50A^2^ − 4.11B^2^ − 5.49C^2^

**Figure 2:**
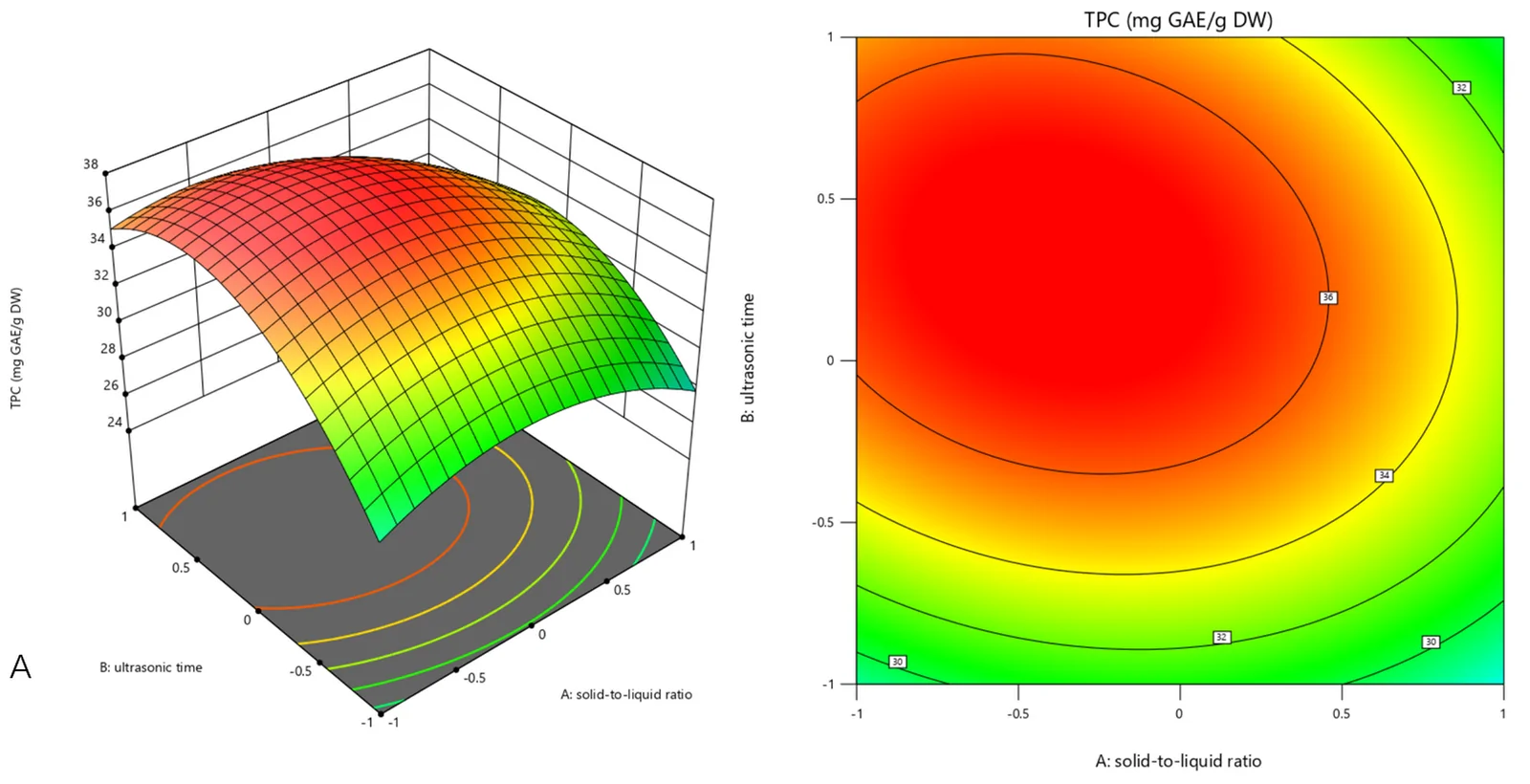

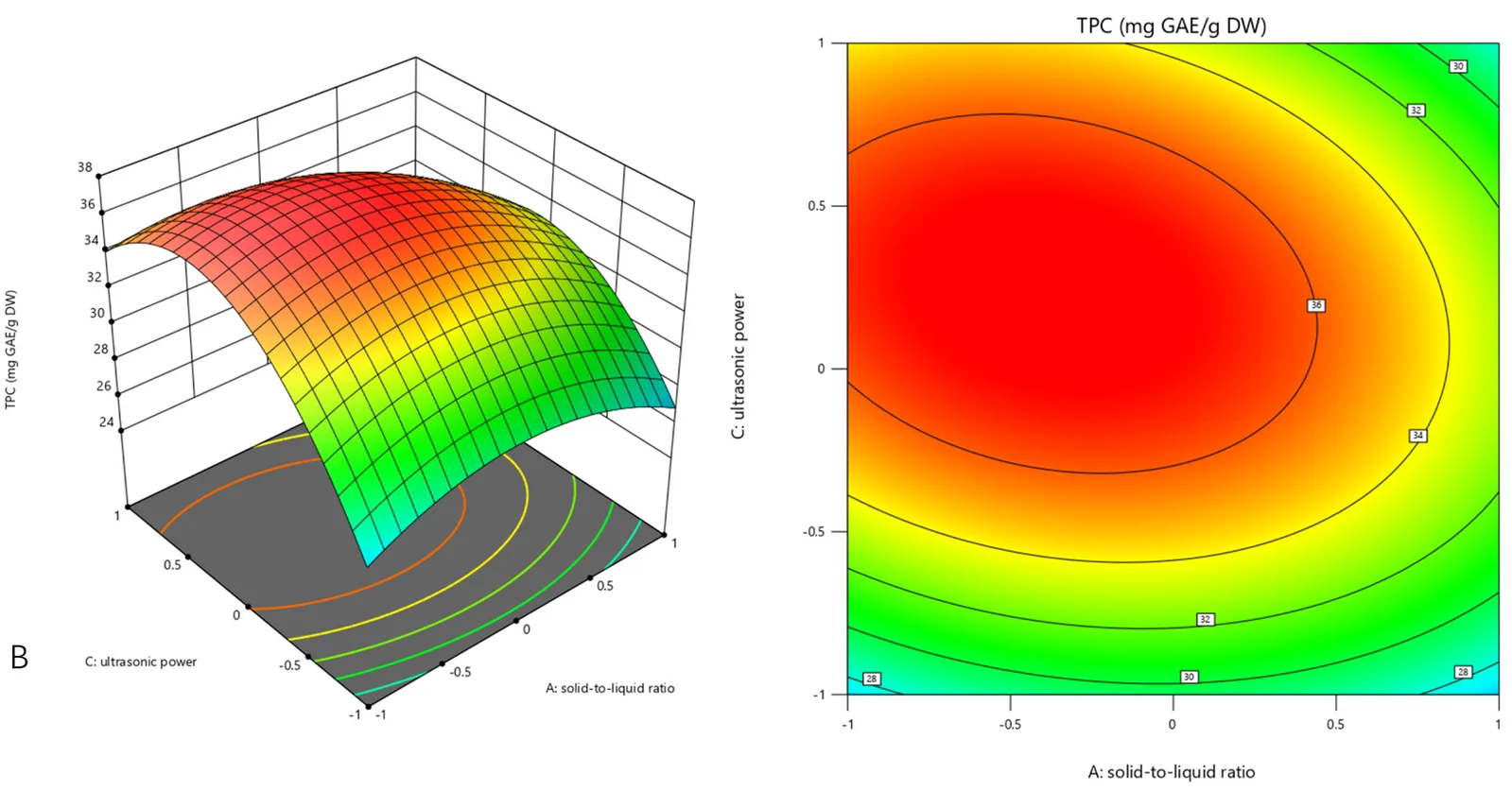

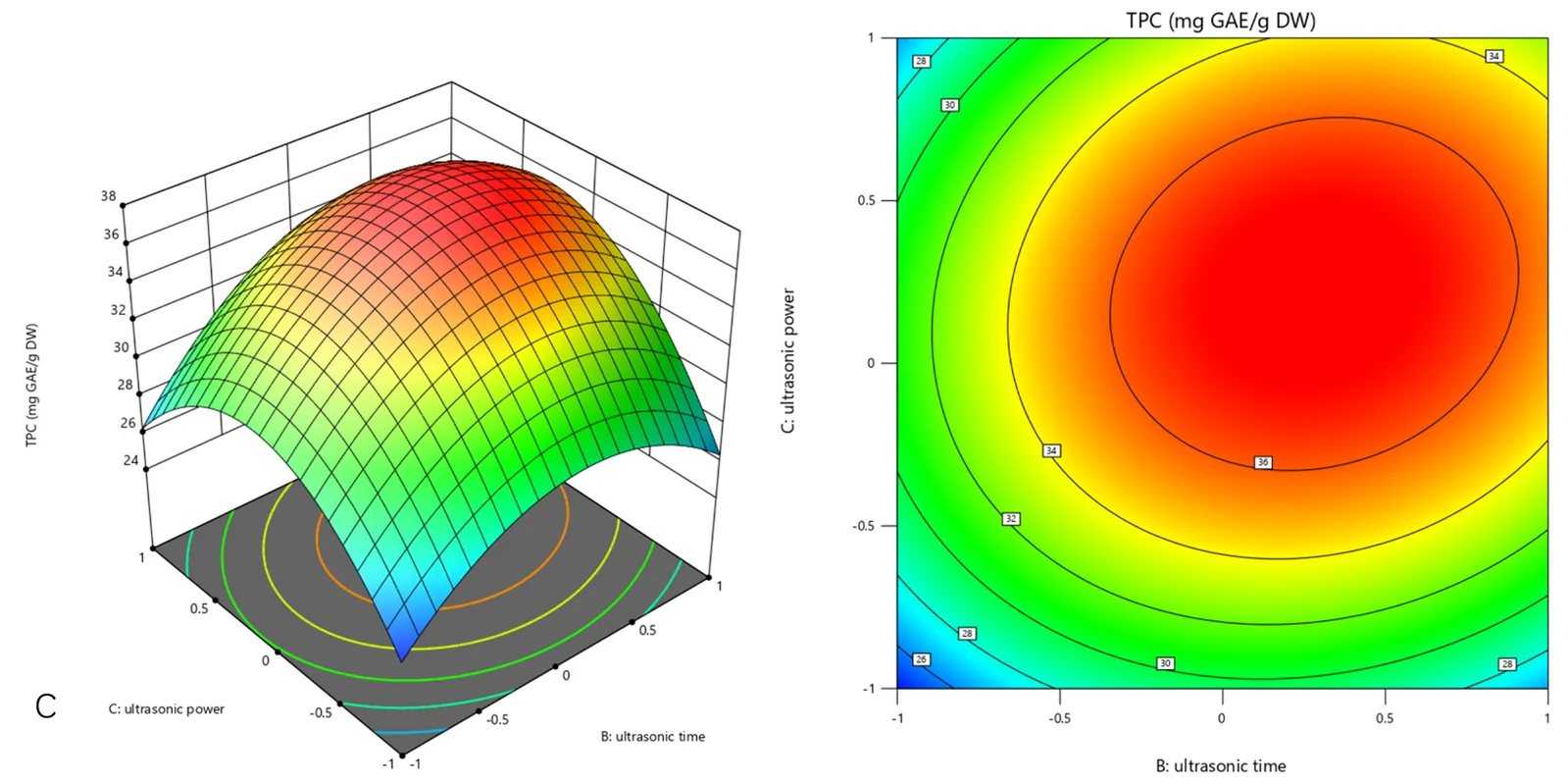
Response surface plots and contour maps showing the influence of each factor on the total polyphenol content. Note: (**A**) solid-to-liquid ratio and ultrasonic time; (**B**) solid-to-liquid ratio and ultrasonic power; (**C**) ultrasonic time and ultrasonic power.

Analysis of variance (ANOVA) (Table 4) revealed that the regression model was highly significant (*p* = 0.0003), with a non-significant lack-of-fit (*p* = 0.0705), indicating excellent predictive reliability. The linear terms (B, C) and all quadratic terms (A^2^, B^2^, C^2^) exhibited highly significant effects (*p* < 0.01) on the extraction yield. The statistical parameters of the fitted response surface model are summarized in Table 5. The model exhibited a high coefficient of determination (R^2^ = 0.9644), indicating that 96.44% of the variability in the response could be explained by the model. The adjusted R^2^ (0.9187) was close to R^2^, suggesting that the model contained no significant redundant terms. The predicted R^2^ (0.8431) was in reasonable agreement with the adjusted R^2^, with a difference of less than 0.2, indicating that the model was not overfitted and possessed adequate predictive capability. The adequate precision value of 12.958 was considerably greater than the minimum desirable value of 4, demonstrating a sufficient signal-to-noise ratio and confirming that the model could be used to navigate the design space. The coefficient of variation (C.V. = 4.07%) was below 10%, reflecting good precision and reproducibility of the experimental data. Collectively, these results indicate that the response surface model was statistically robust and suitable for subsequent optimization and analysis. The model predicted an optimal yield of 38.09 mg GAE/g DW under the conditions of a 1:50.81 (g/mL) solid-to-liquid ratio, 50.24 min, and 118.35 W. For practical operability, these parameters were slightly modified to 1:50 (g/mL), 50 min, and 118 W, yielding an experimental value of 37.93 ± 0.17 mg GAE/g DW. The experimentally obtained value of 37.93 ± 0.17 mg GAE/g DW was close to the model-predicted value of 38.09 mg GAE/g DW, corresponding to a relative deviation of 0.42% and supporting the model prediction near the optimum.The three-dimensional response surfaces and corresponding contour plots (Figure 2).

### 3.3. Scanning Electron Microscopy Observations

**Figure 3:**
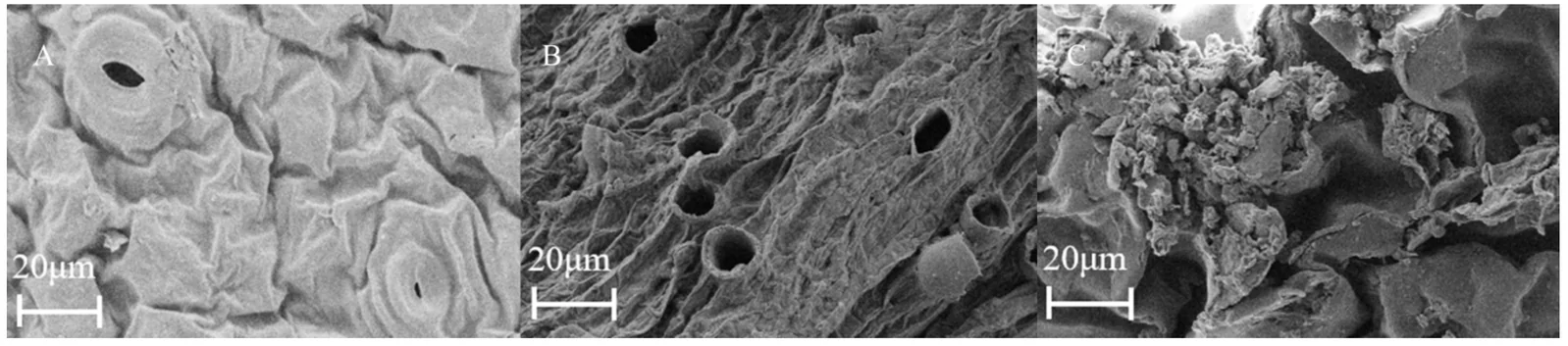
SEM micrographs of tea powder under different treatments: (**A**) untreated; (**B**) water-soaked; and (**C**) water-soaked and ultrasonically treated.

Surface morphologies under the three treatments are shown in Figure 3. Untreated tea powder displayed comparatively smooth and intact surfaces (Figure 3A, 364×), water soaking produced limited surface alteration (Figure 3B, 403×), and UAE-treated powder showed fragmentation, cavities, and collapsed structures (Figure 3C, 382×). These qualitative observations provide morphological support consistent with cavitation-assisted tissue disruption [27], although they do not establish a quantitative relationship between structural damage and polyphenol transfer.

### 3.4. Putative Annotation of Polyphenolic Compounds in the Optimized Zheng’an White Tea Extract

The polyphenolic composition of the optimized Zheng’an white tea extract was investigated using LC–HRMS/MS in both positive- and negative-ion modes. Based on the observed precursor ions, molecular formulae, mass errors, available fragmentation information, and comparison with relevant databases and published data on tea polyphenols, 25 compounds were putatively annotated (Table 6).

The putatively annotated compounds represented several classes commonly reported in tea, including flavan-3-ols, galloylated catechins, procyanidins, flavonols and their glycosides, phenolic acids, and galloylated derivatives [28,29]. Six flavan-3-ols and related gallates were annotated: catechin, epicatechin, (+)-gallocatechin, epigallocatechin, epicatechin gallate, and epigallocatechin gallate. The concurrent annotation of non-galloylated and galloylated flavan-3-ols suggests that structurally diverse catechin derivatives may be present in the aqueous extract.

Several oligomeric or oxidized flavan-3-ol derivatives were also putatively annotated, including procyanidins B2, B3, B5, and C1, as well as theaflavin. Procyanidins are oligomeric flavan-3-ols, whereas theaflavin is an oxidation product derived from catechin-related precursors. Their tentative annotation indicates that the extract may contain flavan-3-ol-related compounds with different degrees of polymerization and oxidation.

The flavonol-related compounds tentatively annotated in the extract included quercetin, quercitrin, rutin, isoquercitrin, kaempferol 3-O-rutinoside, myricetin, and dihydromyricetin. This group comprised both flavonol aglycones and glycosylated derivatives. The annotation of quercetin-, kaempferol-, and myricetin-related compounds is broadly consistent with previously reported polyphenolic profiles of tea [28,29].

Seven phenolic acids and related galloylated compounds were additionally annotated, namely gallic acid, caffeic acid, chlorogenic acid, cryptochlorogenic acid, 4-hydroxycinnamic acid, ellagic acid, and β-glucogallin. The tentative detection of free phenolic acids, chlorogenic acid isomers, and galloylated derivatives further illustrates the chemical diversity of the extract.

Overall, LC–MS supported the putative presence of structurally diverse tea-related polyphenols in the optimized Zheng’an white tea extract, including catechins and related flavan-3-ols, procyanidins, flavonols and their glycosides, phenolic acids, and galloylated derivatives. These findings describe the qualitative polyphenolic profile of the extract. The putatively annotated polyphenols may provide a plausible phytochemical context for the antioxidant activity observed in the subsequent assays.

**Table 6:** Polyphenolic compounds putatively annotated in the optimized Zheng’an white tea extract by LC–HRMS/MS.

No.	Compound	Molecular Formula	InChIKey	RT (s)	Reference *m*/*z*	Observed *m*/*z*	Precursor Ion	Mode	Mass Error (ppm)	Total Score
1	Gallic acid	C7H6O5	LNTHITQWFMADLM-UHFFFAOYSA-N	75.8	153.0182	153.0177	[M−H_2_O+H]+	Positive	3.49	99.9
2	Caffeic acid	C9H8O4	QAIPRVGONGVQAS-DUXPYHPUSA-N	283.3	181.0495	181.0739	[M+H]+	Positive	10.497	98.2
3	Chlorogenic acid	C16H18O9	CWVRJTMFETXNAD-JUHZACGLSA-N	123.3	353.0878	353.0892	[M−H]−	Negative	3.961	99.3
4	Cryptochlorogenic acid	C16H18O9	GYFFKZTYYAFCTR-AVXJPILUSA-N	342.5	355.1024	355.1014	[M+H]+	Positive	2.687	100
5	4-Hydroxycinnamic acid	C9H8O3	NGSWKAQJJWESNS-ZZXKWVIFSA-N	314.9	163.0401	163.0393	[M−H]−	Negative	4.694	100
6	Ellagic acid	C14H6O8	AFSDNFLWKVMVRB-UHFFFAOYSA-N	413.7	300.9990	300.9986	[M−H]−	Negative	1.3	100
7	Beta-glucogallin	C13H16O10	GDVRUDXLQBVIKP-HQHREHCSSA-N	80	331.0671	331.0647	[M−H]−	Negative	6.848	100
8	Catechin	C15H14O6	PFTAWBLQPZVEMU-DZGCQCFKSA-N	221.4	291.0863	291.0857	[M+H]+	Positive	2.098	100
9	Epicatechin	C15H14O6	PFTAWBLQPZVEMU-UKRRQHHQSA-N	300.6	291.0863	291.0858	[M+H]+	Positive	1.755	99.9
10	(+)-Gallocatechin	C15H14O7	XMOCLSLCDHWDHP-SWLSCSKDSA-N	53.9	611.1406	611.1354	[2M−H]−	Negative	8.545	99.6
11	Epigallocatechin	C15H14O7	XMOCLSLCDHWDHP-IUODEOHRSA-N	65.6	305.0667	305.0657	[M−H]−	Negative	3.189	99.8
12	Epicatechin gallate	C22H18O10	LSHVYAFMTMFKBA-TZIWHRDSSA-N	382.3	885.1873	885.1845	[2M+H]+	Positive	3.12	99.6
13	Epigallocatechin gallate	C22H18O11	WMBWREPUVVBILR-WIYYLYMNSA-N	350.2	503.0831	503.0798	[M+HCOO]−	Negative	6.582	100
14	Procyanidin B2	C30H26O12	XFZJEEAOWLFHDH-NFJBMHMQSA-N	213.9	579.1497	579.1494	[M+H]+	Positive	0.511	100
15	Procyanidin B3	C30H26O12	XFZJEEAOWLFHDH-AVFWISQGSA-N	118.6	579.1497	579.1497	[M+H]+	Positive	0.007	100
16	Procyanidin B5	C30H26O12	GMISZFQPFDAPGI-CVJZBMGUSA-N	332.1	579.1497	579.1496	[M+H]+	Positive	0.166	100
17	Procyanidin C1	C45H38O18	MOJZMWJRUKIQGL-XILRTYJMSA-N	331.9	865.1985	865.2038	[M−H]−	Negative	6.093	100
18	Theaflavin	C29H24O12	IPMYMEWFZKHGAX-ZKSIBHASSA-N	412.7	565.1341	565.1541	[M+H]+	Positive	1.903	98.9
19	Quercetin	C15H10O7	REFJWTPEDVJJIY-UHFFFAOYSA-N	430.7	301.0354	301.0338	[M−H]−	Negative	5.231	99.9
20	Quercitrin	C21H20O11	OXGUCUVFOIWWQJ-HQBVPOQASA-N	413.9	449.1078	449.1077	[M+H]+	Positive	0.297	100
21	Rutin	C27H30O16	IKGXIBQEEMLURG-NVPNHPEKSA-N	400.9	611.1607	611.1611	[M+H]+	Positive	0.729	100
22	Isoquercitrin	C21H20O12	OVSQVDMCBVZWGM-ZVIPYZFUSA-N	402.8	927.1837	927.1897	[2M−H]−	Negative	6.506	99.9
23	Kaempferol 3-*O*-rutinoside	C27H30O15	RTATXGUCZHCSNG-QHWHWDPRSA-N	413.7	593.1512	593.1482	[M−H]−	Negative	5.035	98.8
24	Myricetin	C15H10O8	IKMDFBPHZNJCSN-UHFFFAOYSA-N	386.9	319.0448	319.045	[M+H]+	Positive	0.494	100
25	Dihydromyricetin	C15H12O8	KJXSIXMJHKAJOD-LSDHHAIUSA-N	360.1	319.0459	319.0458	[M−H]−	Negative	0.435	100

### 3.5. In Vitro and In Vivo Antioxidant Capacities

**Figure 4:**
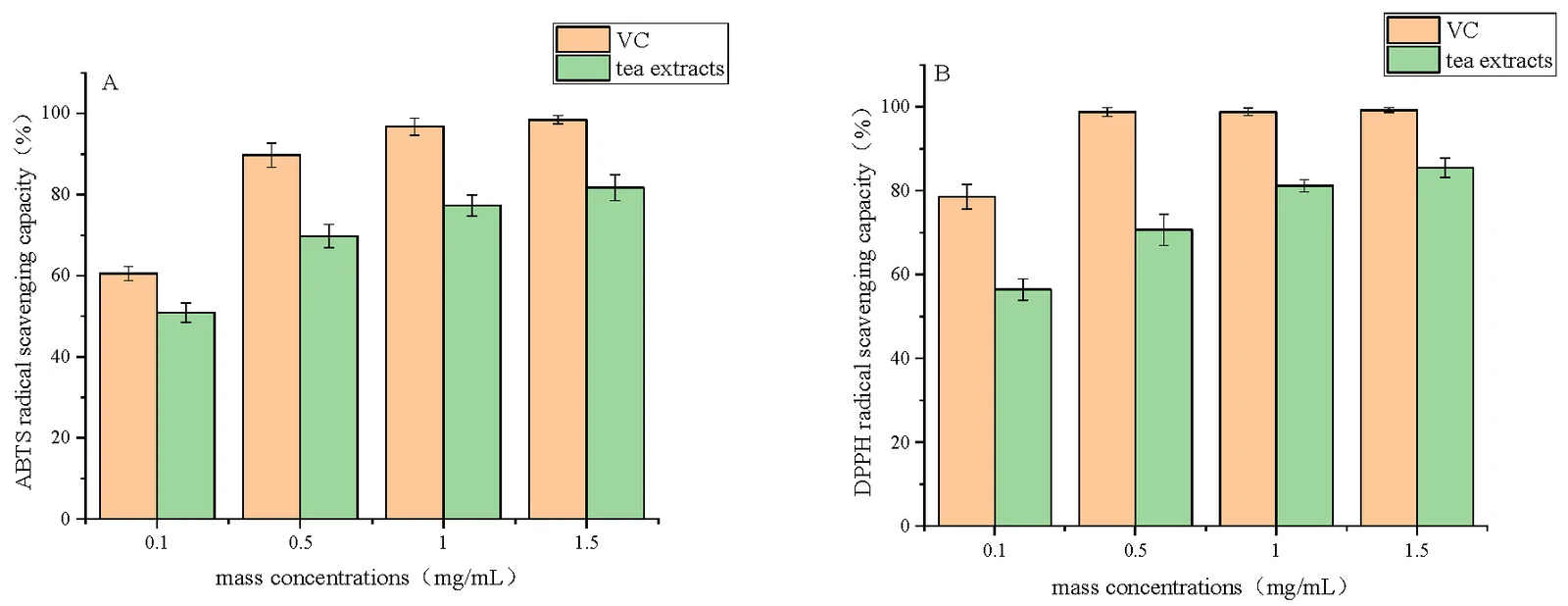
Scavenging activities of tea extracts and the VC reference against ABTS (**A**) and DPPH (**B**) radicals at different concentrations.

The antioxidant potential of the optimized Zheng’an white tea extract was systematically assessed. In vitro, the extract exhibited robust, concentration-dependent scavenging efficacies against both ABTS and DPPH radicals (Figure 4). At a concentration of 1.5 mg/mL, the scavenging rates reached 81.68 ± 3.23% and 85.43 ± 2.31%, respectively. This radical-scavenging response is consistent with antioxidant behavior reported for phenolic structures such as quercetin and cinnamic acid derivatives [30,31]; however, the present qualitative LC–MS data do not establish compound-specific contributions.

**Figure 5:**
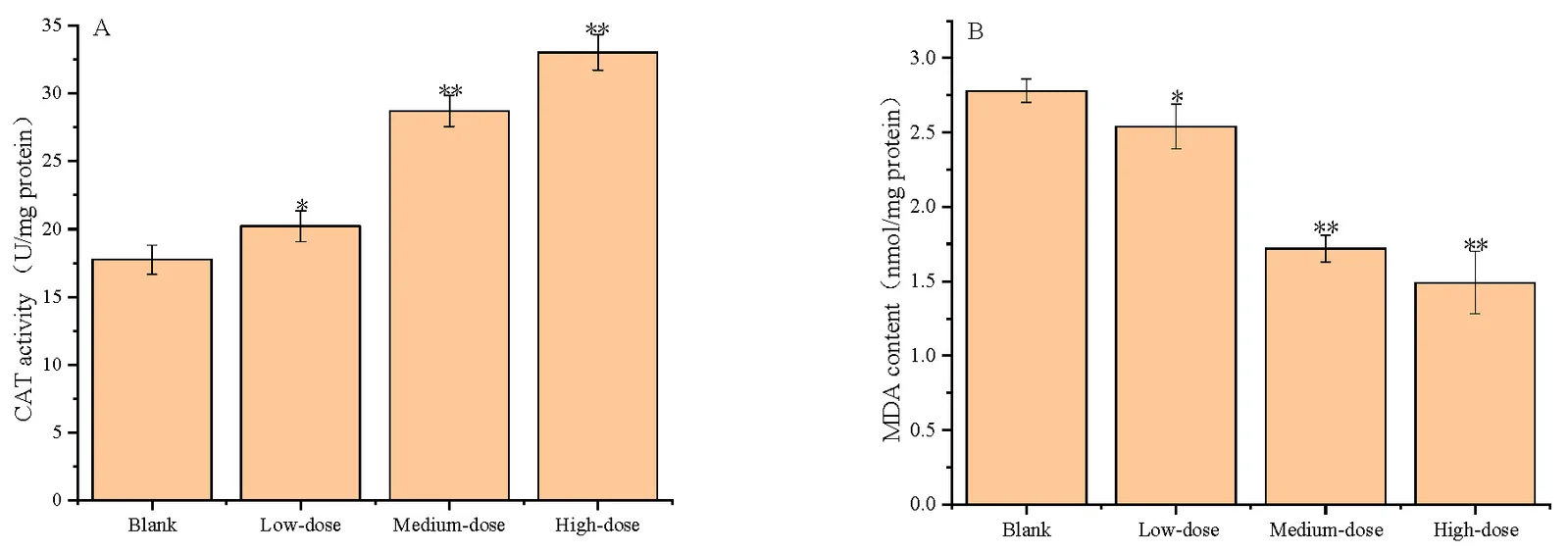
Effects of tea extract on CAT activity (**A**) and MDA content (**B**) in *Caenorhabditis elegans*. *, *p* < 0.05; **, *p* < 0.01.

In the *C. elegans* model, CAT activity and MDA content were used as biochemical indicators of antioxidant defense and lipid peroxidation, respectively. As shown in Figure 5, the 100 μg/mL extract treatment increased CAT activity by 86.03% (to 33.02 ± 1.32 U/mg protein) and decreased MDA content by 46.40% (to 1.49 ± 0.21 nmol/mg protein) relative to the untreated control (*p* < 0.01). These results indicate changes in the measured biochemical endpoints under the tested conditions; they do not by themselves establish a specific signaling mechanism.

## 4. Discussion

This study integrated laboratory-scale process optimization, qualitative microstructural observation, LC–MS annotation, and antioxidant evaluation to examine aqueous UAE of Zheng’an white tea.

The response patterns can be interpreted in terms of both solvent-driven diffusion and acoustic cavitation. Increasing the solvent volume initially maintains a favorable concentration gradient and facilitates diffusion from the tea matrix, whereas further dilution may provide little additional benefit. Likewise, moderate ultrasonic power and duration promote bubble formation and collapse, producing microjets, shear forces, and turbulence that improve solvent penetration and shorten diffusion paths. However, prolonged or overly intense sonication may expose released phenolics to localized thermal and sonochemical effects. The decline observed after 45 min or at higher power in the present single-factor experiments is compatible with this explanation, although the degradation products were not measured here and this mechanism therefore remains inferential.

SEM observations provide qualitative structural support for the extraction response. Compared with untreated and water-soaked particles, UAE-treated particles showed fragmentation, cavities, and collapsed surfaces that are consistent with acoustic disruption and increased solvent-accessible area. Nevertheless, particle-size distribution, porosity, extraction kinetics, and direct structure–yield correlations were not quantified; the microscopy therefore constitutes morphological support rather than direct mechanistic proof.

The putatively annotated features encompassed flavan-3-ols, galloylated catechins, procyanidins, flavonols and their glycosides, phenolic acids, and galloylated derivatives, broadly agreeing with previous LC–MS profiles of tea [28,29]. The present untargeted LC–MS analysis was used only to establish a qualitative profile. Because authentic standards and compound-specific calibration curves were not used, individual polyphenol concentrations and their contributions to antioxidant activity were not determined. Targeted LC–MS/MS analysis with authentic standards is required to quantify individual compounds and evaluate composition–activity relationships.

The concentration-dependent ABTS and DPPH responses indicate that the optimized extract reacted with chemically generated radicals. Similar associations between ultrasound-assisted phenolic recovery and ABTS/DPPH responses have been reported for green tea [32]. These chemical assays cannot establish uptake, metabolism, or antioxidant action in an organism. Related work has also reported improved oxidative-stress resistance in *C. elegans* exposed to dark tea [33]. In the *C. elegans* model, increased CAT activity and decreased MDA content at 100 μg/mL add a biological endpoint, but the present measurements do not identify the causal compounds or signaling pathways.

The in vivo response is directionally consistent with earlier studies of tea constituents in *C. elegans*. Green tea extract has been reported to increase resistance to oxidative stress in the nematode [34], and EGCG increased stress resistance and reduced intracellular hydrogen peroxide in wild-type and stress-related strains [35]. More recently, low-dose EGCG and ECG were shown to increase CAT activity and stress resistance through a mitohormetic response involving AAK-2/SIR-2.1, PMK-1/SKN-1, and DAF-16 signaling [36]. These studies provide biologically plausible contexts for the increased CAT activity observed here, but they do not demonstrate that the Zheng’an white tea extract acts through the same pathways. The extract contains a mixture of catechins, flavonols, procyanidins, and phenolic acids, so its activity may reflect additive, synergistic, or metabolism-dependent effects.

Taken together, the results provide a laboratory-scale basis for considering aqueous Zheng’an white tea extracts in food-oriented antioxidant research. However, the present design does not demonstrate industrial or economic superiority of this specialty, relatively high-value raw material. Lower-value tea grades, pruning material, processing by-products, and spent tea leaves may offer more economical feedstocks; spent tea leaves, for example, can retain extractable polyphenols [37]. Future work should therefore compare raw-material cost, extraction recovery, batch variability, energy demand, and scale-up performance across these alternatives. Freeze-drying was used here to standardize experimental material, but lower-cost drying options also require direct comparison [38]. In addition, because temperature was fixed at 40 °C and was not included as a BBD factor, the reported optimum applies specifically to the tested aqueous UAE system at 40 °C, and optimization across temperatures should be addressed in future work. Additional priorities include matched conventional maceration, solvent-composition studies (including aqueous ethanol), targeted quantification with authentic standards, extract stability during processing and storage, safety and bioaccessibility, and mechanism-focused studies using ROS measurements and pathway-specific *C. elegans* reporters or mutants.

## 5. Conclusions

A three-factor Box–Behnken design was used to optimize aqueous UAE of Zheng’an white tea within the investigated domain. The practical conditions of 1:50 g/mL, 50 min, and an ultrasonic power of 118 W produced a TPC of 37.93 ± 0.17 mg GAE/g DW. SEM observations were consistent with ultrasound-associated tissue disruption. LC–MS enabled the putative annotation of 25 representative polyphenols belonging to several structural classes. The extract showed concentration-dependent ABTS and DPPH radical-scavenging activity and altered CAT activity and MDA content in *C. elegans* at the tested doses. These findings support further evaluation of aqueous Zheng’an white tea extracts, while matched extraction controls, absolute compound quantification, raw-material economics, scale-up, and mechanistic validation remain necessary.

## Figures and Tables

**Table 1:** Box–Behnken design levels for response surface analysis.

Level	A: Solid-to-Liquid Ratio (g/mL)	B: Ultrasonic Time (min)	C: Ultrasonic Power (W)
−1	1:40	30	80
0	1:60	45	110
1	1:80	60	140

**Table 2:** Liquid chromatographic conditions.

Time (min)	Flow Rate (mL/min)	Mobile Phase B
0.0	0.3	2%
1.0	0.3	2%
5.5	0.3	100%
14.0	0.3	100%
14.1	0.3	2%
16.0	0.3	2%

**Table 3:** Box–Behnken experimental design and results for tea polyphenol content.

Run	A	B	C	TPC (mg GAE/g DW)
1	0	1	1	32.47
2	1	0	1	27.13
3	−1	0	−1	27.42
4	1	0	−1	26.86
5	0	−1	−1	24.32
6	1	1	0	30.56
7	0	−1	1	25.79
8	0	0	0	36.35
9	−1	−1	0	29.17
10	−1	1	0	35.55
11	0	1	−1	26.45
12	0	0	0	37.14
13	1	−1	0	28.34
14	0	1	0	34.23
15	0	0	1	36.25
16	0	0	0	36.87
17	−1	0	1	33.22

**Note:** TPC is the average value obtained from three independent extractions.

**Table 4:** Analysis of variance for the response surface model.

Source	Sum of Squares	df	Mean Square	F Value	*p* Value	Significance
Model	303.32	9	33.70	21.09	0.0003	**
A: Solid-to-liquid ratio	19.44	1	19.44	12.16	0.0102	*
B: Ultrasonic time	37.78	1	37.78	23.64	0.0018	**
C: Ultrasonic power	36.00	1	36.00	22.52	0.0021	**
AB	4.33	1	4.33	2.71	0.1439	
AC	7.65	1	7.65	4.78	0.0650	
BC	5.18	1	5.18	3.24	0.1150	
A^2	26.05	1	26.05	16.30	0.0049	**
B^2	68.26	1	68.26	42.71	0.0003	**
C^2	121.97	1	121.97	76.32	<0.0001	**
Residual	11.19	7	1.60			
Lack of fit	10.87	5	2.17	13.48	0.0705	
Pure error	0.3225	2	0.1612			
Total	314.51	16				

**Note:** * indicates significance at *p* < 0.05; ** indicates significance at *p* < 0.01.

**Table 5:** Summary statistics of the response surface model.

Statistic	Value
Std. Dev.	1.264
Mean	31.066
C.V. %	4.069
R^2^	0.9644
Adjusted R^2^	0.9187
Predicted R^2^	0.8431
Adeq Precision	12.958

## Data Availability

The original contributions presented in this study are included in the article. Further inquiries can be directed to the corresponding authors.

## Abbreviations

The following abbreviations are used in this manuscript:

UAE	ultrasound-assisted extraction
BBD	Box–Behnken design
SEM	scanning electron microscopy
LC-MS	liquid chromatography-mass spectrometry
